# Chronic Non-Suppurative Middle Ear Diseases

**Published:** 1910-11

**Authors:** Geo. H. Cooper

**Affiliations:** Opelika, Ala.


### CHRONIC NON-SUPPURATIVE MIDDLE EAR DISEASES.

DR. GEO. H. COOPER, OPELIKA, ALA.

Are we justified in recommending treatment for chronic catarrhal middle ear diseases, so called?

As it is probably true that one person out of every three has defective hearing in one or both ears, it may seem strange that one should ask if we are justified in recommending treatment for the most common cause of this condition. However, I think the consideration of this question timely since it is no uncommon occurrence to hear non-medical men say that treatment for increasing deafness is of no value, and, recently, I have heard general practitioners of medicine with wide experience, say that it was their observation that patients suffering from chronic inflammation of the middle ear heard less and less each year, whether they received treatment or not.

I don't think I exaggerate when I say that the profession generally believes that treatment of this condition is very unsatisfactory. Last week a patient came to me for treatment of increasing deafness, and made the statement that he had been advised several years ago by a well-known physician that treatment would avail him nothing. He was not treated, and since that time has lost all useful hearing in the left ear, and much of the acuteness of the right ear. The loss of hearing in this case has been due to chronic catarrhal inflammation of the eustachian tube and middle ear. If treatment is valuable in these cases, this patient has been injured by the advice given him several years ago, as it was responsible for him not seeking further for relief until many permanent changes had taken place in the middle ear. This case is not unique by any means.

There is an agreement among those who have made a special study of the ear that there are non-suppurative ear diseases which they are unable to benefit, and Politzer predicts that the advanced stages of astosclinasis of the middle ear will always remain beyond the ability of the otologist to reach. On the other hand, otologists firmly contend that there are many nonsuppurative ear diseases—the majority, in fact—that are improved by treatment, and some of them stopped entirely.

Chronic non-suppurative middle ear disease is responsible for the majority of cases of deafness, and most forms of this disease are amenable to treatment. The disease is amenable to treatment because the causes underlying it may often be removed, and if the changes produced in the tympanum are not too far advanced, good hearing may be restored. In many cases the treatments have to be repeated from time to time because we cannot remove the causes, or prevent their return, owing to climatic conditions and habits of living which seem beyond our control. The causes are any conditions which bring about acute catarrhal attacks of the nose, throat and ear, and anything which will tend to make these acute attacks chronic. These causes are local and general; the local, such as obstructions of the nose and throat, sometimes receive the entire attention of the treatment, to the exclusion of the general causes.

Toxines circulating in the blood often seem to have a predilection for the mucous membranes of the head, causing more or less irritation there. Some of these toxines are due to improper action of the skin and digestive tract, intestinal auto-intoxication not infrequently playing a large part in the congestion of these membranes Otologists are realizing in the treatment of this condition, that while no let-up in attention to local causes may be permitted, that more attention must be given to the possible general causes.

Herein, I think, will lie success in the treatment of many cases, which otherwise would have to be counted among the unsatisfactory ones.

Every patient presenting himself for treatment for deafness is entitled to a thorough examination, in order to determine the causes underlying and the seat of the disease in the ear. If the causes are such that they may be removed, and if the seat of the disease is the middle ear, treatment is of great value. If the disease is located in the internal ear, we are able to do little. Usually the seat of the disease may be located with accuracy. A few distinguishing features are as follows: in middle ear changes occur in the drum membrane; in internal ear diseases, the membrane is unaffected. In middle ear diseases, hearing is worse during a "cold in the head;" in internal, no increase is noticed. Paracusi Willissii—the hearing better in a noisy place, is pathognominic of the middle ear disease; in internal ear disease hearing is better in a quiet place. Speaking roughly, the low tones are not heard well in middle ear diseases, while in internal, the low tones are heard better than the higher ones.

As to the treatment of middle ear disease, the first attempt is to reach the cause in the throat and nose and general system. Anything causing a congestion of the naso-pharynx must be removed. If the eustachian tube is closed, it must be opened with air or vapors blown through the catheter.

"Will report case."

Age 55; has suffered with increasing deafness for the last fifteen years. He says he has always been a catarrhal subject. Several years ago he consulted as to treatment for his increasing deafness, and was told that treatment would do him no good. He has recently been unable to hear ordinary conversation. Treatment from time to time for 8 or 10 months improved his hearing, so that he is able to hear his minister preach, something he has been unable to do for some time. He is much pleased with treatment, and there is no question but that treatment would have been worth more to him had it been instituted earlier in his middle ear disease. The results in such cases go to show that all cases of increasing deafness do not get worse each year in spite of treatment. At the same time I expressly disclaim the desire to give the impression that all cases treated give such good results.

It is probably superfluous to say that the earlier treatment is begun the better the results. The time of election for beginning treatment, in many cases, is in childhood, for it is during this stage of physical development that the foundation for most cases of chronic catarrhal Otitis Media is laid.

So I naturally conclude that the proper treatment for chronic catarrhal Otitis Media is not so unsatisfactory in results as is commonly supposed, and it is a mistake to advise patients suffering from increasing deafness that nothing can be done to relieve them.

*Discussion of the paper of Dr. Geo. H. Cooper, Opelika, Ala.*—

Dr. Hugh M. Lokey, Atlanta, Ga.—One of the best short, concise papers I ever heard. I agree with him, in that chronic deafness can be cured. If not entirely cured, it can be so checked as to retain the hearing the patient has at the time the doctor first sees him. I believe that in thirty years we will see very few chronic catarrhal ears, because so much of this is due to diseased adenoids and tonsils. I want to ask the doctor if he has ever used the Seigle Otoscope?

Dr. Cooper, in closing.—In answer to the doctor's question will say, I have used the Seigle Otoscope, and in some cases got good results, and in some, none whatever. My selection of this subject was to bring out before the profession the discouragement the laity have in taking treatment for this trouble, not only the laity, but many general practitioners discourage treatment.

